# Sodium Monitoring in Older Inpatients on Antidepressant Therapy, a Clinical Audit and Service Development Project

**DOI:** 10.1192/bjo.2026.11677

**Published:** 2026-06-30

**Authors:** Huda Khan, Aine Butler

**Affiliations:** 1 National Forensic Mental Health Services, Dublin, Ireland; 2 North Dublin Mental Health Services for Older Persons, Dublin, Ireland

## Abstract

**Aims:**

Hyponatremia is a well-recognised adverse effect associated with several classes of antidepressants, particularly selective serotonin reuptake inhibitors (SSRIs) and serotonin–noradrenaline reuptake inhibitors (SNRIs). Older adults are at increased risk due to age-related physiological changes in renal function, reduced homeostatic reserve, polypharmacy, and the presence of multiple comorbid medical conditions. In the Psychiatry of Old Age inpatient setting, undetected hyponatremia can result in significant clinical consequences, including acute confusion, increased risk of falls, seizures, and overall increased morbidity and length of hospital stay. National and international prescribing guidance, including the NICE guidelines and the Maudsley Prescribing Guidelines, therefore recommend regular serum sodium monitoring in high-risk populations, particularly following the initiation or dose adjustment of antidepressant medication. The aim of this audit was to evaluate compliance with recommended sodium monitoring standards within the Psychiatry of Old Age inpatient unit.

**Methods:**

A retrospective clinical audit was conducted involving all currently admitted inpatients aged 65 years and older on the Psychiatry of Old Age unit who were initiated on antidepressants or experienced antidepressant dose changes during their admission. Data were collected using a standardized audit tool through review of electronic health records, prescription charts, and the laboratory information system. Variables recorded included patient demographics, antidepressant type and dosage, dates of antidepressant initiation or dose escalation, dates of serum sodium measurements, and documentation of abnormal sodium results with associated clinical management.

**Results:**

All patients included in the audit were prescribed antidepressant medication, most commonly mirtazapine and venlafaxine. Baseline urea and electrolyte (U&E) testing was completed for all patients at the time of admission, demonstrating good compliance with admission assessment standards. However, follow-up monitoring within the recommended two to four weeks following antidepressant initiation was documented in only 50% of patients. Antidepressant doses were increased during admission in 50% of patients, yet follow-up U&E monitoring within four weeks of dose escalation was performed in only 16% of cases, indicating a significant gap in adherence to guideline recommendations.

**Conclusion:**

Following presentation of the audit findings to the multidisciplinary team, a quality improvement intervention was implemented. Reminder stickers were introduced into each patient’s file to prompt clinicians to arrange timely sodium monitoring following antidepressant initiation or dose changes. Educational discussion also took place to reinforce guideline awareness and highlight the clinical risks associated with hyponatremia in this population. A second cycle of the audit is currently underway to assess the effectiveness of these interventions and determine whether compliance with sodium monitoring standards has improved.

